# Exploring public genomics data for population pharmacogenomics

**DOI:** 10.1371/journal.pone.0182138

**Published:** 2017-08-03

**Authors:** Kleanthi Lakiotaki, Alexandros Kanterakis, Evgenia Kartsaki, Theodora Katsila, George P. Patrinos, George Potamias

**Affiliations:** 1 Institute of Computer Science, Foundation for Research and Technology, Heraklion, Crete, Greece; 2 Department of Pharmacy, School of Health Sciences, University of Patras, Rio, Patras, Greece; 3 Department of Pathology, College of Medicine and Health Sciences, United Arab Emirates University, Al-Ain, UAE; CNR, ITALY

## Abstract

Racial and ethnic differences in drug responses are now well studied and documented. Pharmacogenomics research seeks to unravel the genetic underpinnings of inter-individual variability with the aim of tailored-made theranostics and therapeutics. Taking into account the differential expression of pharmacogenes coding for key metabolic enzymes and transporters that affect drug pharmacokinetics and pharmacodynamics, we advise that data interpretation and analysis need to occur in light of geographical ancestry, if implications for drug development and global health are to be considered. Herein, we exploit ePGA, a web-based electronic Pharmacogenomics Assistant and publicly available genetic data from the 1000 Genomes Project to explore genotype to phenotype associations among the 1000 Genomes Project populations.

## Introduction

Several studies have demonstrated the genetic underpinnings of inter-individual variability in drug response [1–3], among which the Veterans Administration Cooperative Study implied a key role for pharmacogenomics and geographical ancestry on the basis of the variable drug responses observed, when propranolol and hydrochlorothiazide were administered to African-Americans and Caucasians [4]. Similarly, the administration of BiDil, a drug to treat African-American patients with heart failure has been subject to considerable criticism, when clinical trials showed strong evidence of extreme effectiveness in self-identified African-American patients and at the same time, considerable evidence that the effects were far smaller, if present at all, in Caucasian patients [5]. In a recent publication in the New England Journal of Medicine, Vence and coworkers accurately stated that although self-identified race may correlate with geographical ancestry, it does not predict an individual patient's genotype or drug response and thus, prescribing medications on the basis of race oversimplifies the complexities and interplay of ancestry and drug response [6].

Thus, population based-studies are needed to infer rates and risks for drug inefficacy and/or adverse drug reactions (ADRs) and guide the implementation of pharmacogenomic testing. Indeed, several studies report an extensive variability in genetic variants and gene expression relationships associated with pharmacogenes in humans, when ethnic and/ or racial groups are considered [7–10]. Approximately one-fifth of new drugs approved in the past six years demonstrated differences in exposure and/or response across racial/ethnic groups, leading to population-specific prescribing recommendations. Ramamoorthy and coworkers reviewed several such cases [11].

Genomic variation describes naturally occurring differences among individuals of the same species resulting from non-random mating, genetic drift, physical distribution, or migration. Genomic variation is typically discovered by sequencing individual genomes and comparing reads to a reference human genome (http://www.ncbi.nlm.nih.gov/projects/genome/assembly/grc/human/). More than 80 million variant sites in the human genome have been discovered so far, as registered and curated by the Genetic Variation Program (http://www.genome.gov/10001551). These variant sites include single nucleotide polymorphisms (SNPs), insertions and deletions (indels) as well as other structural variants. In total, the genetic architecture of quantitative traits and/or genotype to phenotype correlations, meaning whether numerous rare variants of large effect or common variants of very small effect account for human genome variation is still arguable [12].

Paving the way to Pharmacogenomics (PGx)-guided decision-making in the clinic, current findings indicate that pharmacogenes are of fundamental importance when drug responses need to be predicted [13–15]. Genomic variants in drug-metabolizing enzymes and transporters have been linked to inter-individual differences in the efficacy and toxicity of many medications [16] and thus, PGx studies empower drug discovery and tailored-made theranostics [17]. Moreover, next generation sequencing (NGS) has created unprecedented opportunities towards the analysis of whole genomes by obtaining a full picture of one’s variome [18,19]. Inter-ethnic differences in allele frequencies of genes encoding for proteins involved in drug absorption, distribution, metabolism, excretion and toxicity (ADMET) have been reported in several studies.

Considering that drug-metabolizing profiles, defined by the distribution of drug metabolizing enzyme variants, differ significantly among genetically inferred clusters, “…*it is not only feasible but a clinical priority to assess genetic structure as a routine part of drug evaluation*…” [20]. FDA strongly advises that PGx assessment in early-phase clinical studies is necessary [21], pinpointing that most early-phase clinical trials in a setup of limited ethnic diversity. Herein, we exploit ePGA, a web-based electronic Pharmacogenomics Assistant and publicly available genetic data from the 1000 Genomes Project to explore genotype to phenotype associations among the 1000 Genomes Project populations with the aim to support decision-making for drug development and global health.

## Towards Population Pharmacogenomics (PPGx)

According to Priority Medicines for Europe and the World 2013 report (http://www.who.int/medicines/areas/priority_medicines/en/), the term ‘stratified medicine’, might be more accurate than the term “personalized medicine”, or its newer term “precision medicine”–the delivery of “the right drug to the right patient” and the minimization of ADRs, the current focus being the biomarker-based stratification of patient populations. Besides the race to discover PGx biomarkers over the last years, a common note in the PGx community is the lack of implementation of the PGx knowledge into routine clinical practice under an individualized framework [22]. Among the several obstacles that hold up the translation of PGx knowledge into improved health, “*the lack of prospective genotype-directed pharmacogenomic randomized clinical trials validating treatment algorithms”* as mentioned in the Pharmacogenomics Research Network (PGRN) Translational Pharmacogenetics Program is of fundamental importance to us [23].

Although the stratified randomization of clinical trials (RCT) based on genotypes raises ethical, legal, and practical concerns, it remains a promising approach to transform current clinical trials and enable clinical pharmacogenomics. Pereira and coworkers [24] discuss the rationale for genotype-based RCT in cardiovascular disease showing that by identifying population groups that are likely to be more susceptible to a potential ADR, drug development companies can eliminate the huge cost and length of clinical trials. Following such a population-based PGx approach, genomic variation that relates to ADR risks may be identified, turning information growth to knowledge growth (translational research, drug development, clinical applications, patient awareness and education) [25,26].

A common example of genetic diversity in drug response among populations comes from tamoxifen, a drug that is used to treat breast cancer patients. Tamoxifen’s pharmacological activity depends on CYP2D6 enzyme function. About 6–10% of Europeans are poor CYP2D6 metabolizers (PM) compared to <1% of East Asians [27]. In this context, Li and coworkers reported that many ADMET genes are highly differentiated across continental regions and found numerous signals of recent positive selection [28].

In this work, we show how current PGx discoveries, as deposited in state-of-the-art knowledge bases (e.g., PharmGKB), can be translated into findings which, not only enhance the underlying drug development pipelines, but also facilitate the identification of population differences in drug response/toxicity events. For this, we investigated inter-individual and population-based differences in the allele frequencies of known pharmacogenes in the 1000 Genomes Project Phase III (1kG-p3) dataset, consisting of 2,504 individuals from 26 different populations and 5 ancestral groups. We focused on inter-individual differences at the group (population or ancestral) or molecular level (genotype or haplotype) and identified those ADMET genes that show greater phenotypic variation among different populations.

## Materials and methods

### Study populations

A total of 2,504 individuals from 26 populations with ancestry from different parts of the world (5 ancestral groups) were included in this study. Their genotypes were extracted from the VCF files of Phase 3 variant calls of the 1000 Genomes Project (http://www.1000genomes.org/) sample collection (1kG-p3). Details on the population data can be found in [29].

### Pharmacogenomics database

PharmGKB [30] was established in 2000 as one of the first ‘post-genomic’ databases for the description, storage and curation of genotype and phenotype data from PGx studies. PharmGKB in alliance with CPIC (Clinical Pharmacogenetics Implementation Consortium) [31] and PGRN (PharmacoGenomics Research Network; pgrn.org) [32] presents the most comprehensive resource on pharmacogenes (PGx genes), their variations, the pharmacokinetic and pharmacodynamic pathways of interest as well as their effects on drug-related phenotypes, while it freely offers PGx clinical annotations and drug dossing guidelines. Furthermore, PharmGKB provides haplotype information for a list of genes. The term “haplotype” refers to a cluster of allelic variants (SNPs, insertions, deletions, etc.) that are co-inherited because of their complete or strong linkage disequilibrium coupled to their chromosomal proximity to one another. PharmGKB does not define haplotypes. Instead, PharmGKB collects and curates information on haplotype definitions for specific genes from different sources, such as the haplotypes that define cytochrome P450 (CYP) alleles, which are derived from the “The Human Cytochrome P450 (CYP) Allele Nomenclature Database” [33]. Currently, The Human Cytochrome P450 (CYP) Allele Nomenclature Database user can have access to the nomenclature for the polymorphic alleles of 29 CYP enzymes. *CYP2B6*, *CYP2C9*, *CYP2C19* and *CYP2D6* genes are the most polymorphic, all with a high number of functionally different alleles [34].

In the case of no centralized resources or an entity responsible for reconciling haplotypes, PharmGKB attempts to collect gene haplotype information from relevant published studies (with links to the respective PMIDs). If a study does not provide a name for a haplotype, PharmGKB uses sequential numbering to provide each haplotype with a name in order to distinguish amongst the different gene haplotypes (see: https://www.pharmgkb.org/page/faqs#—What is a PharmGKB haplotype?).

As of April 2015, we downloaded the so-called PharmGKB “translation tables” with haplotype data for 69 PGx genes that engage 727 different variants, noted as “PGx variants” hereafter, involved in 764 haplotypes. PharmGKB considers the first haplotype listed in each table as the reference, namely the “wild-type” haplotype for that set. More details on those translation tables and their underlying processing for their use in genotype to phenotype translations are found in [35].

### ePGA

ePGA [35] is a web-based electronic Pharmacogenomics Assistant (https://www.epga.gr/) that provides personalized genotype-to-phenotype translation, linked to state-of-the-art clinical guidelines. ePGA's translation service matches individual genotype profiles with PGx gene haplotypes and infers the corresponding diplotype and phenotype profiles, accompanied with summary statistics. ePGA offers two main services: “Explore” and “Translate”. The ePGA explore service is a user-friendly browser to PGx gene-variation-drug-metabolizing status associations and links to corresponding dossing guidelines and/or clinical annotations. The ePGA translation service matches individual genotype profiles with PGx gene haplotypes to infer the corresponding diplotype and phenotype profiles, accompanied with respective summary statistics. As a result, we leveraged ePGA’s services to develop exploratory methods and visualization tools to easily and efficiently explore current PGx findings in publicly available genetic data.

## Results and discussion

### Pharmacogenomics variant analysis among populations

To eliminate any inconsistencies arising from the use of different human genome references, we analyzed the PGx variants of interest on the basis of their rs ID that matched both the reference and variant alleles as noted in PharmGKB and Ensembl. We always consider the allele designated in PharmGKB tables as the variant allele. We also used Ensembl’s BioMart [36], accessed with the BioMart R package [37] that uses the most recent GRh38 human genome assembly release (housed by GRC/ Genome reference Consortium).

**Fig 1:**
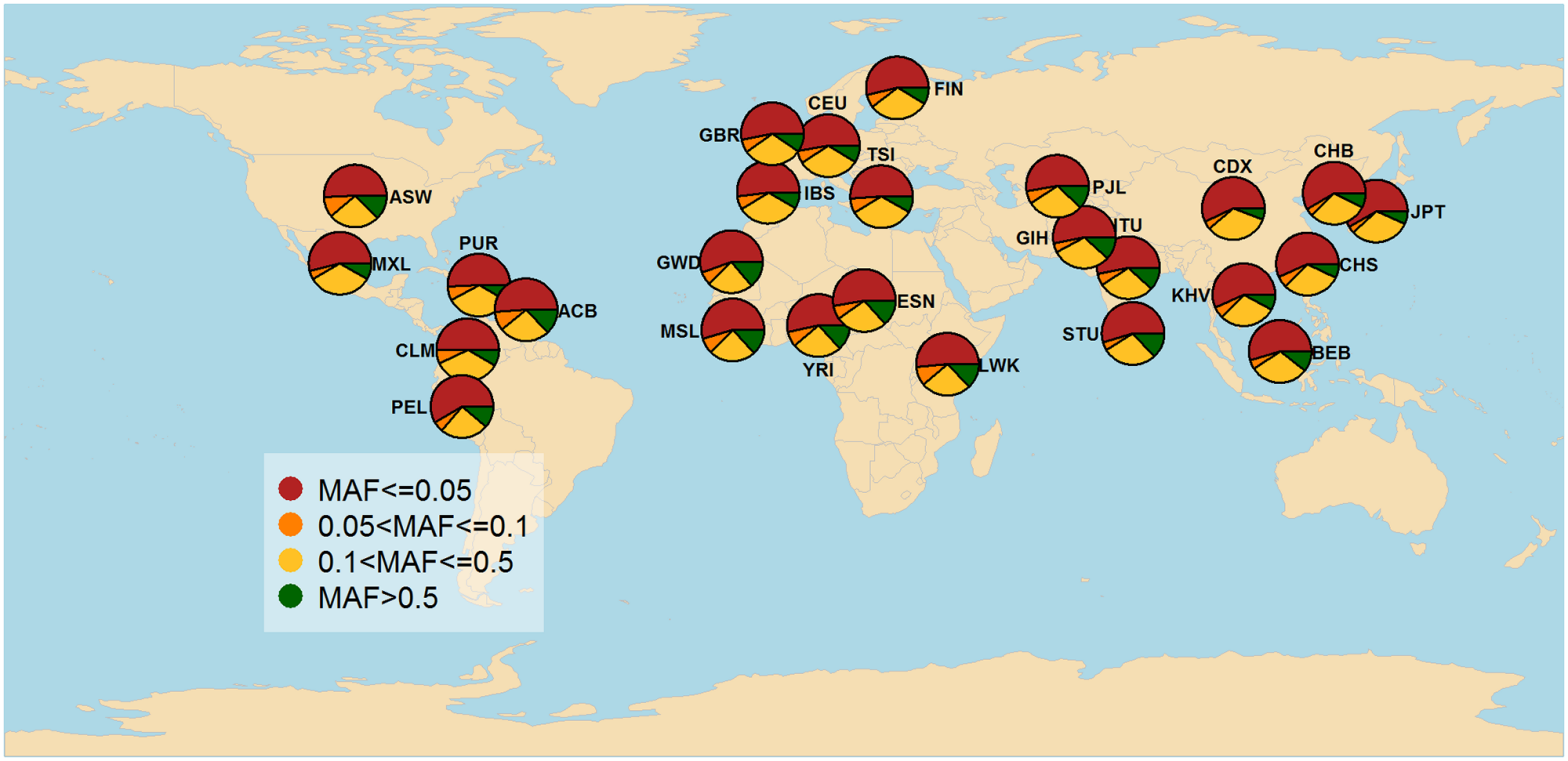
Geographic MAF distribution of 501 PGx variants found in the 1000 Genomes project populations.

We only found 501 PharmGKB variants (out of the 727, as of April 2015) in the 1kG-p3 dataset shared across 26 populations. These variants are involved in 328 different haplotypes (Tables A-E in S1 File). We denote as “variant allele” or simply “variant”, the allele related to the PGx effect and by Minor Allele Frequency (MAF) its occurrence across each population. In Fig 1 we show the MAF distribution across all 26 populations. It is noticeable that most PGx variants in all populations are rare variants (MAF< = 0.05).

Using BioMart we extracted chromosome location, gene names (as HGNC symbols) as well as SIFT [38] and PolyPhen [39] scores. SIFT predicts whether an amino acid substitution affects protein function (an amino acid substitution with a SIFT score of less than 0.05 is characterized as deleterious). PolyPhen predicts the possible impact of an amino acid substitution on the structure and function of a human protein (an amino acid substitution with a PolyPhen score greater than 0.15 is possibly damaging). Both predictions rely on whether or not an amino acid is conserved in the protein family, which can be indicative of its importance to the normal function or structure of the expressed protein. There are also some approaches that combine various tools to predict the effects of non-synonymous SNPs or databases, which are specific to a series of genes relevant to the biology of cancer. Herein, we are interested in capturing the overall picture of the PGx effects on the related protein function. Therefore, we adopt a simple classification and color-coding scheme for the functional consequences of PGx variants:

1. PGx variants with SIFT scores greater or equal to 0.05 and PolyPhen scores lower or equal to 0.2 are labeled as “*benign*” and colored green;
2. PGx variants with SIFT scores lower than 0.05 and PolyPhen scores greater than 0.2 are labeled as “*damaging*” and colored dark-red;
3. PGx variants for which either SIFT or PolyPhen scores are not available are labeled as “*unknown*” and colored grey;
4. PGx variants whose SIFT and PolyPhen scores lead to controversial characterizations are labeled as “*ambiguous*”.

**Fig 2:**
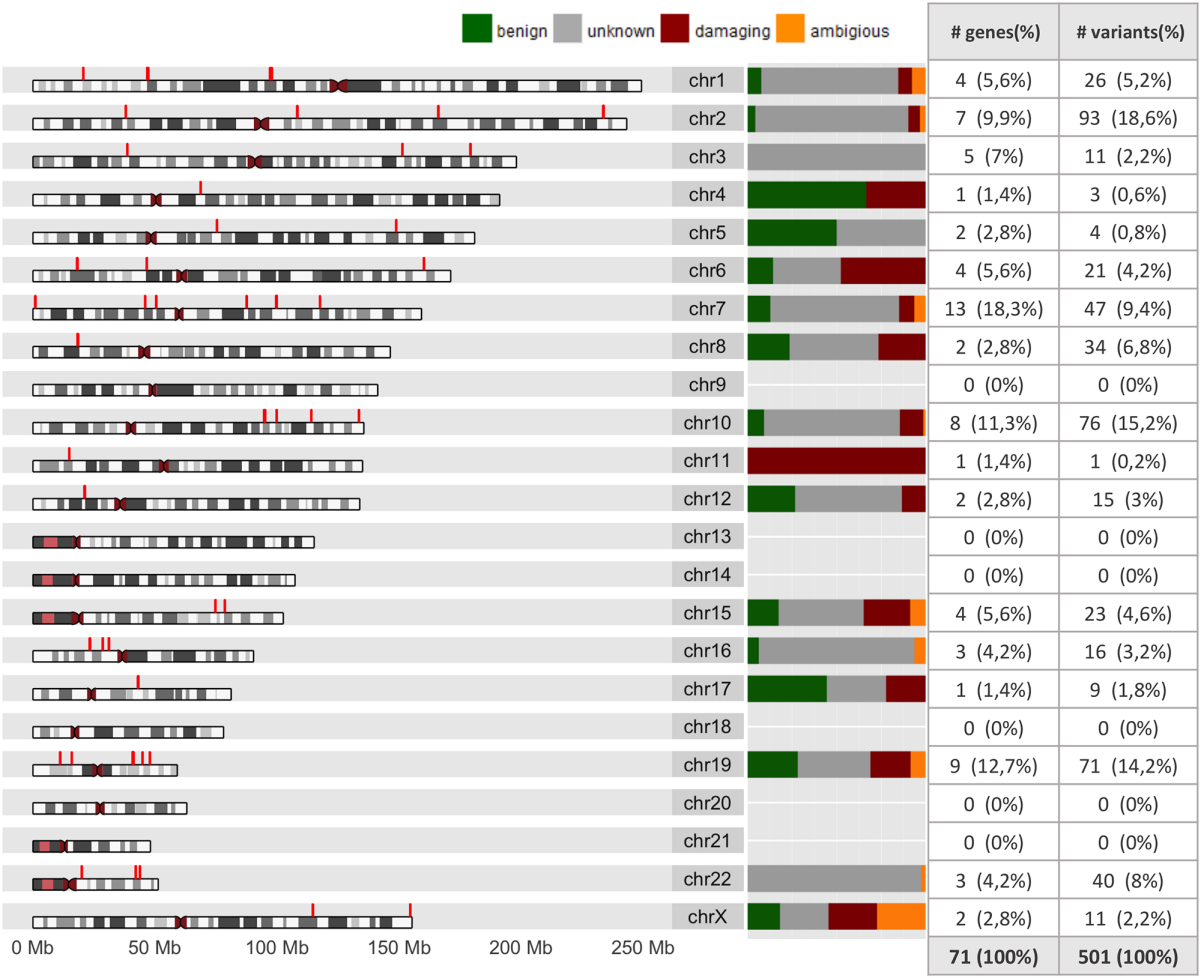
Chromosomal location and functional consequence distribution per chromosome of the PGx variants. The accompanied table shows the PGx variants and genes located in each chromosome.

SIFT and PolyPhen scores were calculated using Ensembl’s variant effect predictor (VEP) tool [40]. In Fig 2 we show the chromosomal location and distribution of the inferred (according to the aforementioned classification schema) functional consequences of PGx variants per chromosome.

We noticed that there are no PGx variants of interest on chromosomes 9, 13, 14, 18, 20 and 21. Most 1kG-PGx variants (93 in total) are located on chromosome 2, followed by chromosome 10 and 19, with 76 and 71 PGx variants, respectively. We also noticed that even though only one variant has been discovered so far on chromosome 11, this refers to rs61495246, which is a missense variant that associates with Vitamin D hydroxylation-deficient rickets. On chromosome 6, 10 out of the 21 variants were predicted as possibly damaging, while 7 of them were associated with *TPMT* and 3 with *SLC22A1*. All possibly damaging PGx variants were found with MAF<0.05 in the 1kG population.

**Fig 3:**
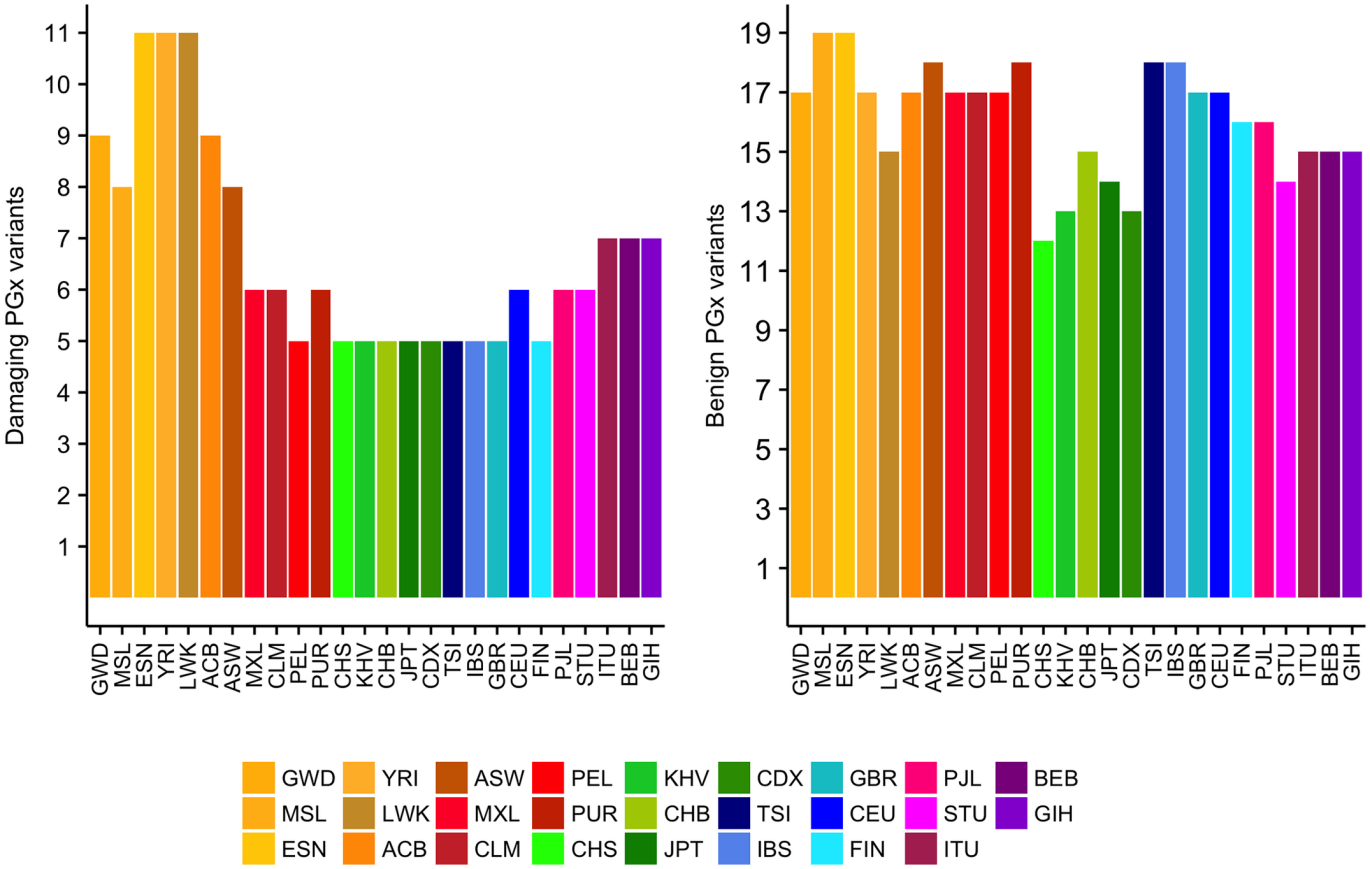
Distribution of common (MAF≥0.1) possibly damaging (left) and benign (right) PGx variants across 1kG populations. These are African/AFR (GWD, MSL, ESN, YRI, LWK, ACB, ASW), Ad Mixed America/AMR (MXL, CLM, PEL, PUR), East Asian/EAS (CHS, KHV, CHB, JPT, CDX), European/EUR (TSI, IBS, GBR, CEU, FIN), South Asian/SAS (PJL, STU, ITU, BEB, GIH) ancestral groups.

Overall, we found 72 possibly damaging and 69 benign PGx variants according to the aforementioned classification. In Fig 3 we show the distribution of common (MAF≥0.1) possibly damaging (left) and benign (right) PGx variants across the 26 1kG populations. We notice that populations of African ancestry exhibit the greatest number of common possibly damaging PGx variants.

Ramos et al. [41] analyzed an ‘actionable’ subset of 42 variants identified by the FDA as important pharmacogenomic biomarkers, which are also listed in various drug labels and have supporting evidence of clinical utility in PharmGKB. We found 37 of those markers in the 1kG-p3 dataset. When referring to any of those variants throughout this work, we use italics to designate them.

**Fig 4:**
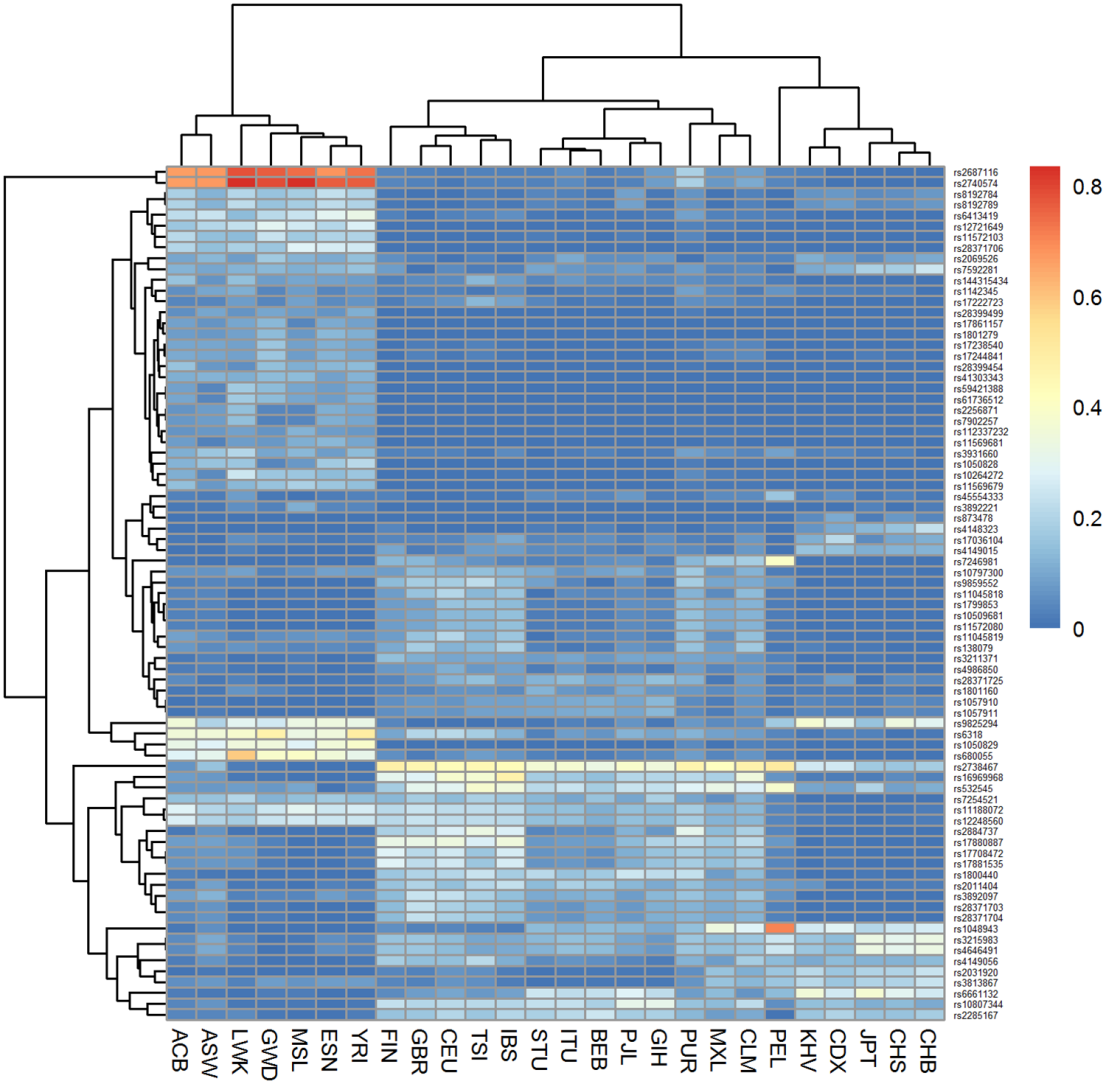
Minor Allele Frequency heatmap for 79 PGx variants that are common (MAF> = 0.1) and rare (MAF< = 0.005) in at least one 1kG population.

It is known that common variants are shared across populations, whilst rare variants are often restricted to individual or closely related populations [29]. We searched for variants that are very rare (MAF< = 0.005) or common (MAF> = 0.1) in at least one population. We found 79 such variants and plotted their MAF heatmaps. As shown in Fig 4, these PGx variants can be indicative of ancestral groups, except from the PEL population which cannot be clustered to any ancestral group, when adjusting the dendrogram according to the minimum height for ancestral clustering.

Notably, rs1048943 (*CYP1A1*) has a MAF of 0.7 in PELs, being a missense variant and thus, a potential prognostic marker for survival outcome after docetaxel plus capecitabine chemotherapy in metastatic breast cancer patients [42]. Furthermore, rs7246981, a missense variant associated with (*CYP2F1*) is found in high frequency (MAF = 0.6) only in PELs. rs2687116 (an intronic variant in the *CYP3A4* gene) and *rs2740574* (an upstream variant in the *CYP3A4* gene) are found in exceptionally higher frequencies in all populations of African ancestry. *rs2740574* may be associated with an increased likelihood of methadone fatality [43].

Nine of the 79 variants reported herein (rs1050828, *rs1057910*, *rs1142345*, rs11569679, *rs11572103*, *rs1801279*, rs2256871, *rs28399499*, rs4986850) are characterized as possibly damaging and therefore, pharmacogenomic-guided drug administration is necessary among populations. All nine of them are rare variants with global MAFs<0.05 except for *rs11572103*, which has a slightly higher global MAF (MAF = 0.055). Overall, we identified three common (MAF>0.1) damaging PGx variants across all populations studied; rs72466456 (*NAT2*), rs3740066 (*ABCC2*) and rs1799966 (*BRCA1*).

**Fig 5:**
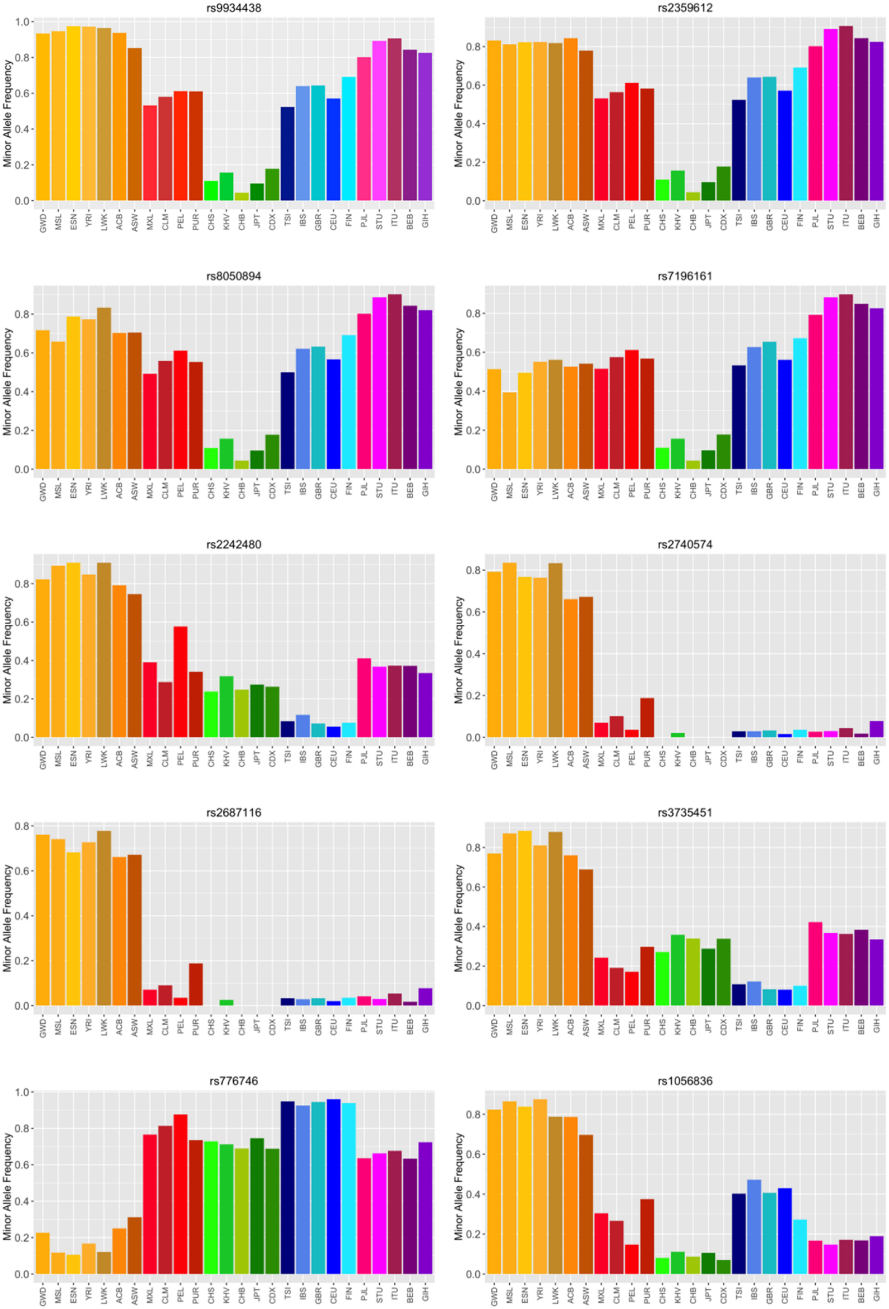
Minor Allele frequency distribution for the ten most differentiated PGx variants among 1kG ancestral groups.

We define ΔMAF as the difference between the maximum and the minimum MAF of any ancestral group. In Fig 5 we show the MAF distribution among populations of ten PGx variants that exhibit ΔMAF greater than 0.7 among ancestral groups.

The first four PGx variants (rs9934438, rs2359612, rs8050894 and rs7196161) are *VKORC1* variants. *VKORC1* encodes for vitamin K epoxide reductase complex subunit-1, a small transmembrane protein of the endoplasmic reticulum, which plays a major role in the vitamin K pathway and is the target protein of warfarin. These variants exhibit high MAF in all populations, but the East Asians. In addition, four *CYP3A4* variants (rs2242480, *rs2740574*, rs2687116, rs3735451) are extensively found in their alternative form only in individuals of African Ancestry.

**Fig 6:**
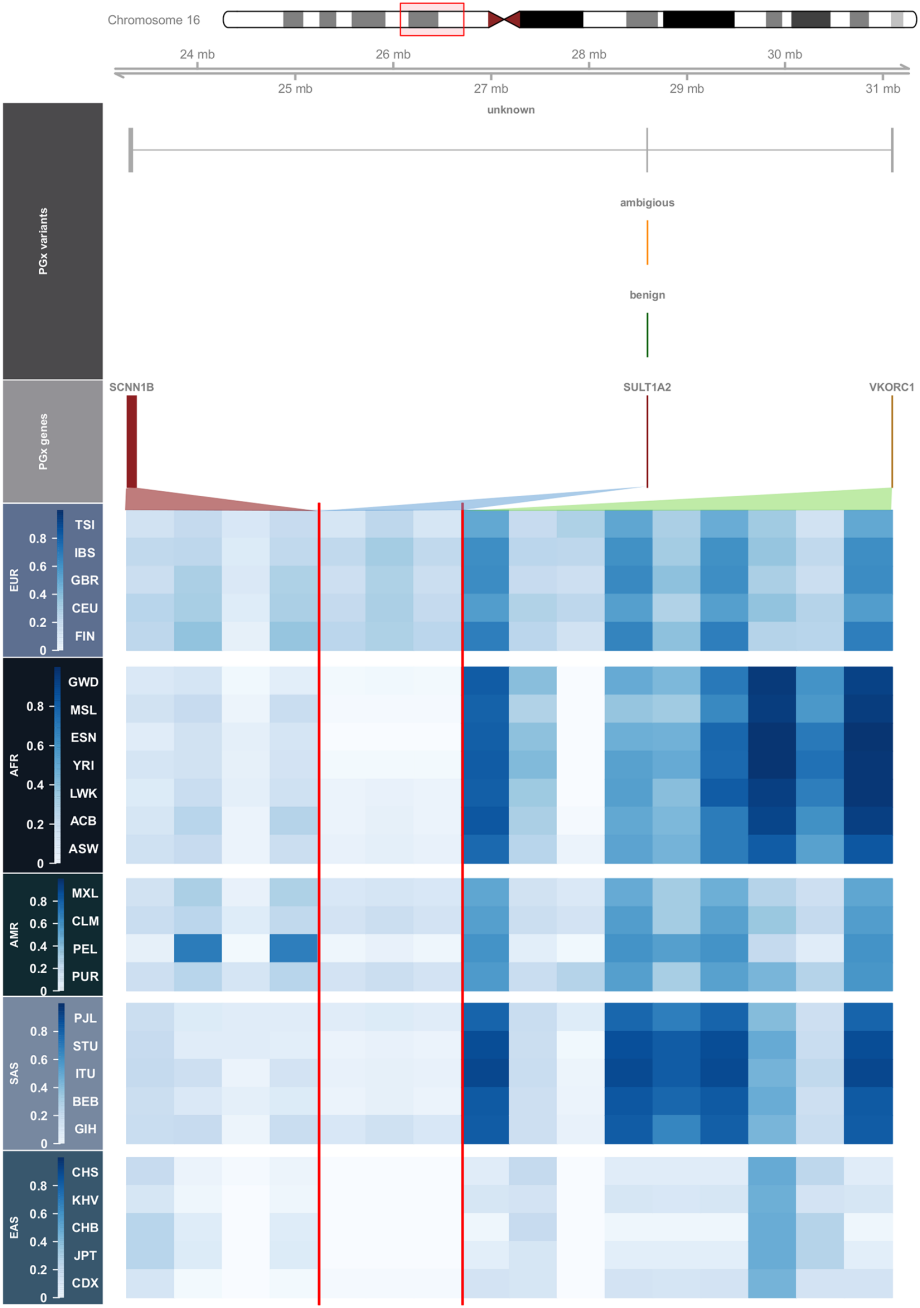
Genomic annotation plot combined with PGx variant MAFs in 1kG populations.

To explore PGx-related population differences visually, we compiled genomic information together with MAFs in a single plot using the GViz R package [44]. In Fig 6 we first plot the chromosome ideogram, where current genomic location is indicated by a red box, followed by a genomic axis. The red box starts at the location of the first PGx variant and ends at the location of the last PGx variant. On the PGx variants track we provide information on the functionality of the PGx variants according to the aforementioned classification (‘*unknown’*, ‘*ambiguous’*, ‘*benign’*, ‘*damaging’*). In the chromosome 16 example, we can see that all three genes (*SCNN1B*, *SULT1A2*, *VCORC1*) share unknown variants, whilst *SULT1A2* relates to both benign and ambiguous variants. On the PGx genes track we show the genes of interest and their PGx variants which are located on chromosome 16 (their width is analogous to the location of their variants). Subsequently, we provide a heatmap of the MAFs on all populations grouped by their ancestry, according to which the darker the color code, the greatest the MAF is, while the vertical red line separates the variants of different genes.

### Pharmacogenomics haplotype analysis among populations

Given that PGx markers are discovered using genotyping arrays, most PGx variants are “tags” for haplotypes on which the directly functional variants reside. However, as already shown, several PGx variants are population-specific and thus, some tests might perform well only on those populations that follow the haplotype structure that these tests underlie. Samwald and coworkers [45] examined the accuracy of such haplotype inferences across different 1kG populations and found that current haplotype definitions are incomplete when the actual 1kG data are considered for a large fraction of samples.

**Fig 7:**
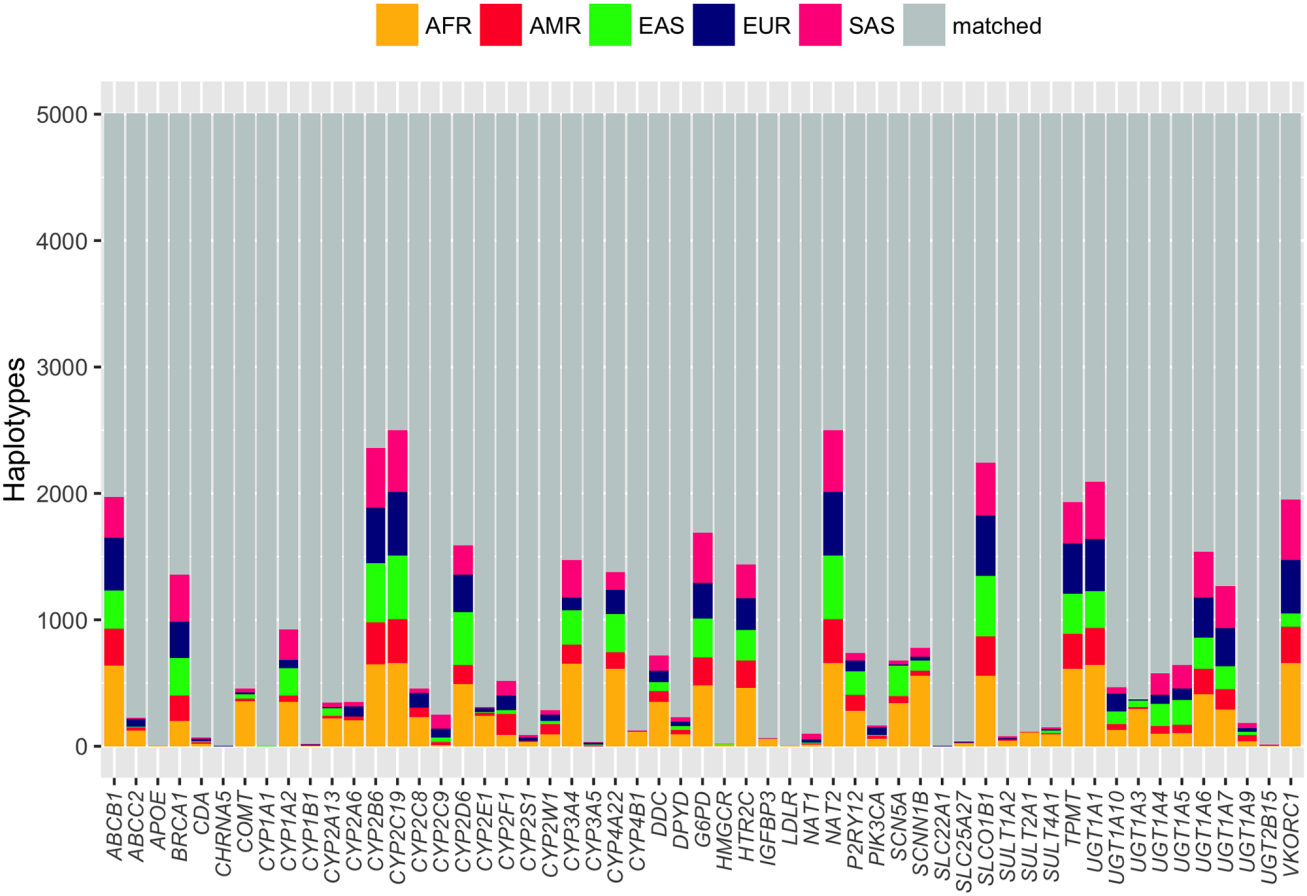
Individual haplotypes that did not match to any known haplotype per gene. Colours represent the five ancestral groups. Grey fills indicate haplotype matches.

Fig 7 depicts the percentage of individual haplotypes that did not match to any given haplotype. Indeed, we found unmatched haplotypes in 55 out of 65 genes. Genes with the least haplotype coverage in 1kG data include *NAT2*, *CYP2C19*, *CYP2B6*, *SLCO1B1*, *UGT1A1*, *ABCB1*, *VKORC1* and *TPMT*.

Since a PGx haplotype consists of one or more variants in their alternative form, we define as “haplotype variability” the number of variants in their alternative form that a haplotype enables. By definition, a “wild type” haplotype has zero alternative variants. At first, we investigated the number of PGx variants in their alternative form that were included in a haplotype. We found that 91% of haplotypes include up to three PGx variants and just eleven haplotypes (1 in *CYP2C19*, 3 in *CYP4A22*, 1 in *SULT4A1* and 6 in *UGT1A3*) include a relatively large (>5) number of alternative PGx variants. In particular, UGT1A3*2c and CYP2C19*2C include 11 such variants.

Herein, most of the individuals were matched to “wild type” haplotypes. However, 20–30% of individuals were matched to haplotypes of high variability, since 33% of all individuals were matched to the UGT1A3 *2a haplotype. We report that UGT1A3 *2a refers to 9 different PGx variants in their alternative form and is correlated with increased atorvastatin lactonization, which may affect its lipid-lowering effect [46]. Additionally, 19% of the individuals tested matched to the CYP4A22 *12A haplotype, which refers to the alternative form of 6 PGx variants, while 14% of all individuals were matched to the CYP4A22 *15 haplotype (8 PGx variants). Noteworthy, the genomic variants and haplotype structures of the *CYP4A22* gene have been extensively studied in a Japanese population [47]. Last, 22% of 1kG-p3 individuals were matched to the SULT4A1 *5 haplotype, which refers to the alternative form of 8 PGx variants. *SULT4A1* codes for a brain-specific sulfotransferase that is believed to be involved in the metabolism of neurotransmitters. There is a surprising amount of heterogeneity in the genetic diversity of the different *SULT* genes. *SULT4A1* is the most highly conserved gene through evolution, sharing over 97.5% sequence identity between mouse, rat, chimp and human *SULT4A1* coding sequences [48]. In Table 1, we report the Haplotype Frequencies (HAFs) per ancestry group for four haplotypes that exhibit the highest variability and significant percentage match to the different ancestral groups. The global (among all individuals) haplotype frequency is depicted in the last column.

**Table 1:** Matching distribution of individuals per ancestry group for the haplotypes with the highest variability and significant percentage match.

Gene Haplotype	Variability	AFR	AMR	EAS	EUR	SAS	Global
CYP4A22 *12A	6	0.16	0.13	0.25	0.11	0.26	0.17
CYP4A22 *15	8	0.01	0.10	0.30	0.08	0.24	0.12
*SULT4A1 #5*	8	0.34	0.15	0.07	0.21	0.26	0.20
UGT1A3 *2a	9	0.48	0.35	0.12	0.25	0.41	0.27

**Fig 8:**
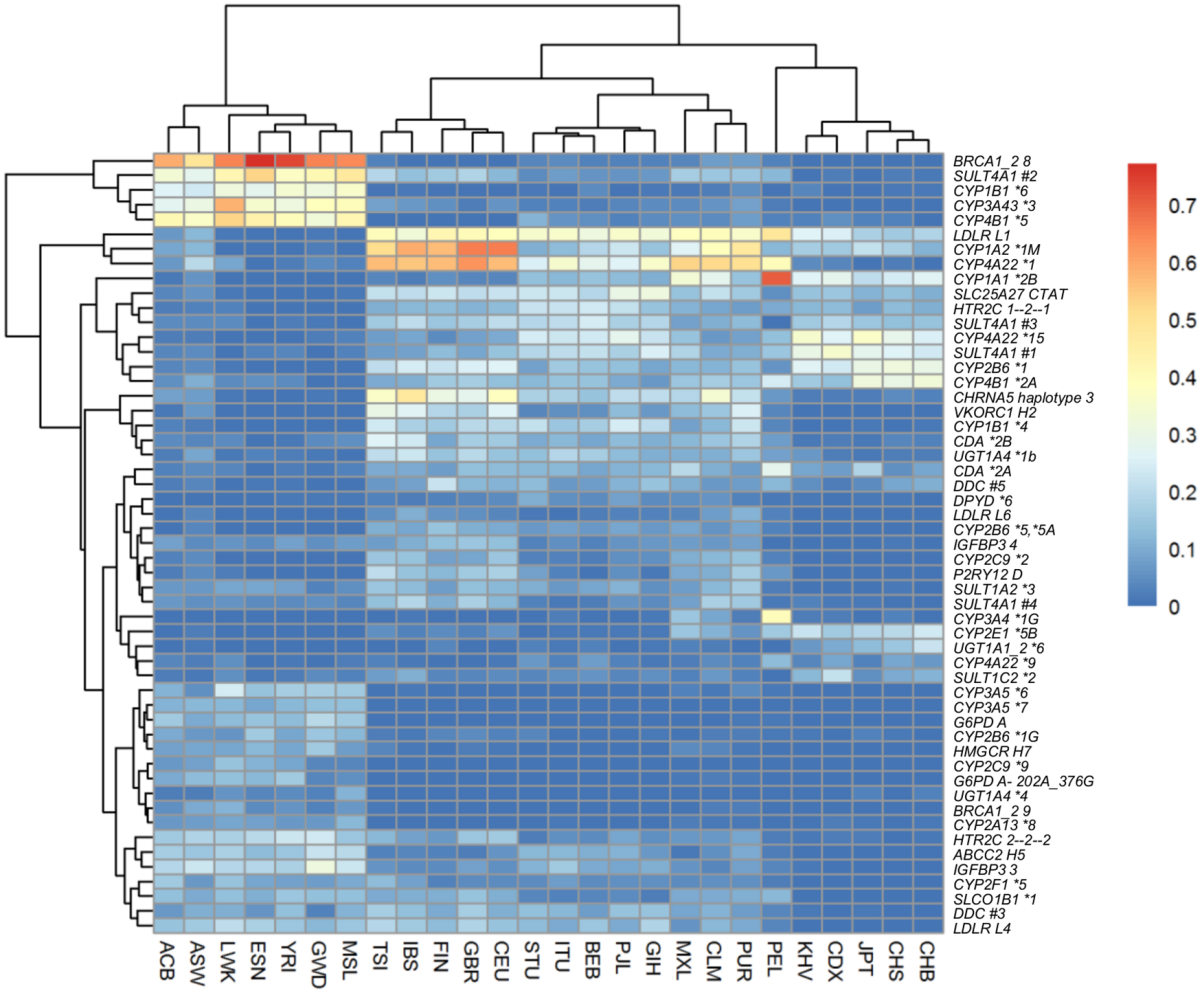
Haplotype Frequency (HAF) heatmap for 53 PGx haplotype that are common (HAF> = 0.1) and rare (HAF< = 0.005) in at least one 1kG population.

We also discovered 53 haplotypes with common (HAF ≥0.1) and rare (HAF ≤0.005) haplotype frequencies in at least one population. As shown in Fig 8, we noticed that highly differentiated haplotypes result from either a single population difference, such as in the CYP1A1*2B for PELs, or more often from populations of common ancestry.

From the 328 haplotypes that match at least one population, most of them (40%) matched to all populations, as opposed to the 11% that are found in a single population. In total, 130 haplotypes match to individuals from all populations. The % percentage of haplotypes that match all populations is 37%, as opposed to the low frequency of haplotypes (2%) that match just one population.

### Pharmacogenomics phenotype analysis among populations

**Fig 9:**
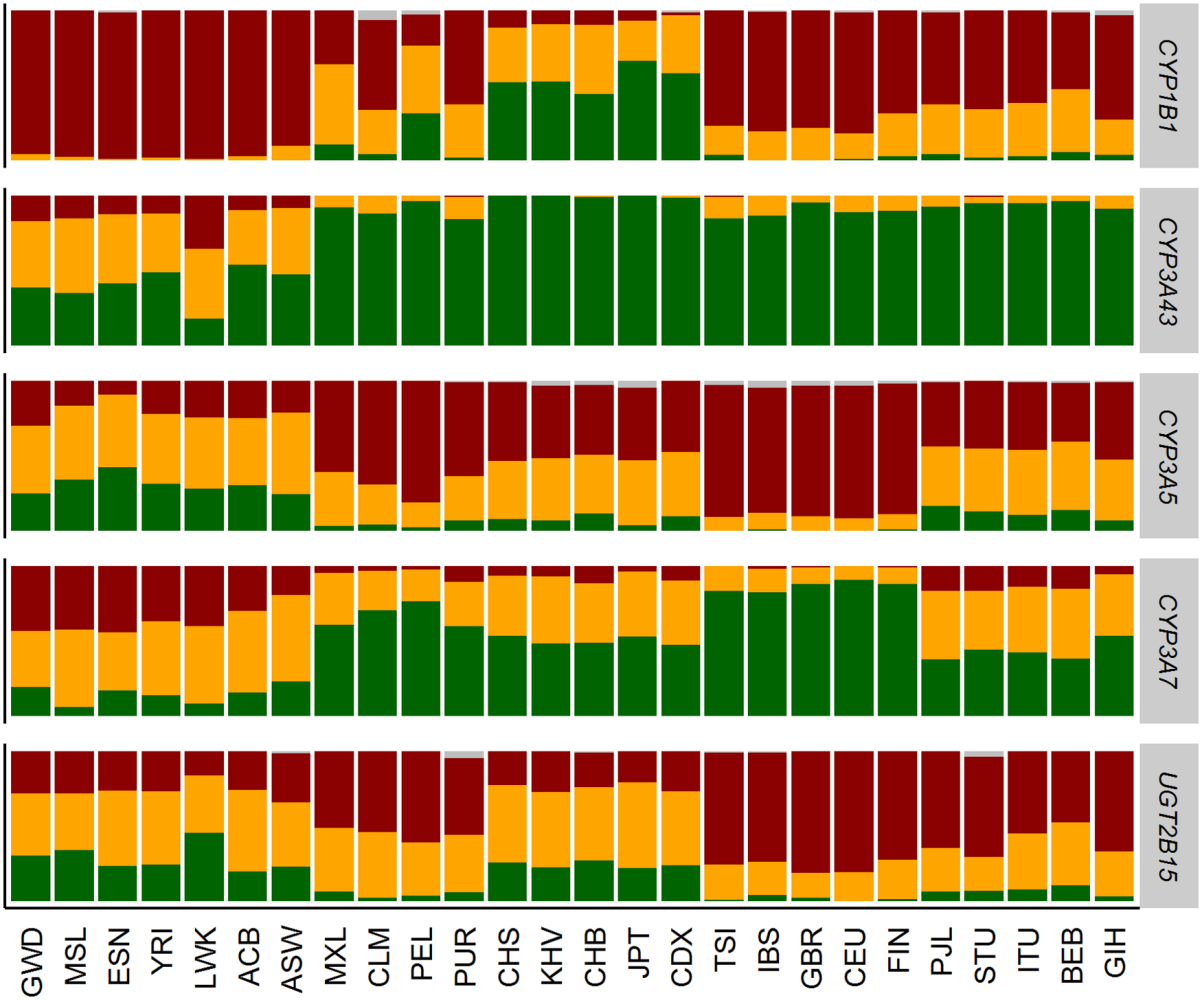
Distribution of three phenotypes (WT/WT-green, WT/V-orange, V/V-red) for 5 highly covered genes with high phenotypic difference among 1kG populations.

Next, we defined three PGx phenotypes as inferred by the related genotype profiles and adopted a color classification schema to indicate them: ‘WT/WT’ (wild-type/wild-type) in green, where both alleles match ‘WT’ haplotypes; ‘WT/V’ (wild-type/variant) in yellow, where one of the alleles match a ‘WT’ and the other one a ‘V’ haplotype; and ‘V/V’ (variant/variant) in dark red, where both alleles match ‘V’ haplotypes. We assume that wild type/wild type (WT/WT) vs. wild type/variant (WT/V) vs. variant/variant (V/V) haplotypes lead to normal, intermediate and abnormal drug metabolizing statuses, respectively. In Fig 9 we show the phenotypic distribution for 5 genes, for which a phenotype was assigned in most (> = 90%) individuals (one or more phenotypes is found with frequency 80% or greater in one population and 20% or lower in another).

High phenotypic differences appear mainly among ancestral groups. It is noticeable that although most populations are probably *CYP3A43* WT/WT drug meta, African ancestry populations and especially LWKs are mostly WT/V or even V/V drug metabolizers. By using ePGA’s explore service (https://www.epga.gr/explore/), we can easily find that *CYP3A43* is related to olanzapine, an atypical antipsychotic, approved by the FDA for the treatment of schizophrenia and bipolar disorder. We were unable to find any drugs related to *CYP1B1* and *CYP3A7*. *CYP3A5* is related to 17 different drugs and *UGT2B15* is related to oxazepam and sipoglitazar. The ePGA’s explore service retrieves gene-drug information from PharmGKB.

## Conclusions

The advent of genome sequencing technologies has led to an enormous and rather complex amount of data revealing the vast complexity of our genome architecture. To this end, the Precision Medicine Initiative Working Group of the National Institutes of Health has recommended that the All of Us Research Program (https://www.nih.gov/research-training/allofus-research-program) should enroll participants from diverse social, racial/ethnic, ancestral, geographic, and economic backgrounds, from all age groups and health statuses to broadly reflect the diversity of the U.S. population. To better study and understand our genomic variation, efficient computational tools enriched with exploratory and analysis features that can provide meaningful visualizations for the translation from genotype to phenotype are necessary.

Herein, we demonstrated how we can discover PGx knowledge from the exploratory analysis of ePGA data by exploiting publicly available genetic data. We showed that PGx biomarkers are able to distinguish population groups and thus, can be exploited to design and develop stratified clinical trials. Interesting exploratory findings can be made either at the molecular level (PGx variant- haplotype-phenotype) or by exploring PGx information globally at the ancestral or population level. At the variant level, we found that most PGx variants in all populations are rare variants (MAF< = 0.05). We identified three damaging PGx variants common (MAF>0.1) to all populations and 72 possibly damaging rare PGx variants (mean global MAF = 0.03). Populations of African ancestry were found to have the greatest number of common (MAF>0.1) damaging PGx variants. Notably, 79 PGx variants were very rare (MAF< = 0.005) and/or common (MAF> = 0.1) in at least one population, 9 of which are possibly damaging PGx variants and therefore, pharmacogenomic-guided drug administration across populations seems necessary. At the haplotype level, we found that 91% of haplotypes refer to up to three PGx variants (alternative alleles). 53 haplotypes with frequency greater or equal to 0.1 in at least one population and lower or equal to 0.005 in another varied among populations. The CYP4A22 *15 haplotype which refers to 8 PGx variants in their alternative form was more frequently matched to East Asian populations. At the phenotypic level, we found 5 genes, for which a phenotype was assigned in most (> = 90%) individuals, while one or more phenotypes were found in least 80% in one population and at most 20% in another. High phenotypic differences appeared mainly among ancestral groups.

Herein, publicly available data serve as a model to illustrate the means as well as the findings of interest and as such, of importance. Indeed, we describe how and why, by further exploring publicly available data (1000 Genomes Project), the user reveals pharmacogenomic (PGx) findings in three different levels of information (variant- haplotype-phenotype levels) to ensure data quality towards clinical implementation: a. even though 79 PGx variants differ among populations (MAF> = 0.1 and MAF< = 0.005 in at least one 1kG population), only 46 PGx variants differ among populations when haplotypes are considered (those with HAF > = 0.1 and HAF ≤0.005 in at least one population); b. when phenotypes are considered, only three genes (*CYP1B1*, *CYP3A43* and *CYP3A5*) constitute phenotypes with variable distribution among populations (one or more phenotypes is found with frequency 80% or greater in one population and 20% or lower in another) and c. to the best of our knowledge, this is one of the very few analysis that further support the outcome of Mizzi et al. [49] and Zhou et al. [50] regarding population differences and their implications for clinical PGx. As an example, we report that rs1048943 (*CYP1A1*) has a MAF of 0.7 in PELs (see Fig 4), being a missense variant and thus, a potential prognostic marker for survival outcome after docetaxel plus capecitabine chemotherapy in metastatic breast cancer patients. The CYP1A1 *2B haplotype that includes both rs1048943 and rs4646903 is also found in high frequency only in PELs (Fig 8). However, *CYP1A1* does not show high phenotypic differentiation (as defined in this work) among populations. Such findings suggest that PGx researchers and clinicians should study PGx findings from the genotype to phenotype level and not focus on a single level of information, since a single variant may be extensively variable among populations, yet its effect on the phenotype of interest may be masked or modified by one or more variants.

Ongoing and future work involves the integration of the exploratory and visualization methods developed herein in ePGA services and the extension of those methods to include MutationInfo (http://mutationinfo.readthedocs.io/en/latest/), a python package that extracts the position, the reference and the alternative sequence of a genomic variant and accepts variants in dbSNP rs or HGVS format. We envisage to integrate genotype to phenotype translation, exploration and analysis of pharmacogenomic data into a single tool with the aim to enable population-based pharmacogenomics and catalyze the integration of pharmacogenomics into the clinic.

## Supporting information

S1 File (XLSX)
